# Analysis of Factors Influencing Miners’ Unsafe Behaviors in Intelligent Mines using a Novel Hybrid MCDM Model

**DOI:** 10.3390/ijerph19127368

**Published:** 2022-06-16

**Authors:** Xinping Wang, Cheng Zhang, Jun Deng, Chang Su, Zhenzhe Gao

**Affiliations:** 1 School of Management, Xi’an University of Science and Technology, Xi’an 710054, China; zc@stu.xust.edu.cn (C.Z.); 21202097023@stu.xust.edu.cn (Z.G.); 2 School of Safety Science and Engineering, Xi’an University of Science and Technology, Xi’an 710054, China; dengj518@xust.edu.cn

**Keywords:** intelligent mine, miners’ unsafe behaviors, DEMATEL, MMDE, MCDM

## Abstract

Coal mine accidents seriously affect people’s safety and social development, and intelligent mines have improved the production safety environment. However, safety management and miners’ work in intelligent mines face new changes and higher requirements, and the safety situation remains challenging. Therefore, exploring the key influencing factors of miners’ unsafe behaviors in intelligent mines is important. Our work focuses on (1) investigating the relationship and hierarchy of 20 factors, (2) using fuzzy theory to improve the decision-making trial and evaluation laboratory (DEMATEL) method and introducing the maximum mean de-entropy (MMDE) method to determine the unique threshold scientifically, and (3) developing a novel multi-criteria decision-making (MCDM) model to provide theoretical basis and methods for managers. The main conclusions are as follows: (1) the influence degree of government regulation, leadership attention, safety input level, safety system standardization, and dynamic supervision intensity exert the most significant influence on the others; (2) the causality of government regulation, which is the deep factor, is the highest, and self-efficacy displays the smallest causality, and it is the most sensitive compared to various other factors; (3) knowledge accumulation ability, man–machine compatibility, emergency management capability, and organizational safety culture has the highest centrality among the individual factors, device factors, management factors, and environmental factors, respectively. Thus, corresponding management measures are proposed to improve coal mine safety and miners’ occupational health.

## 1. Introduction

Mining is the industry with the highest risk globally, with accident rates up to 10 times higher than other industries [1]. Coal mine accidents affect people’s lives, property safety and social development, and human unsafe behaviors are the main reason for accidents [2,3]. In China’s coal mining industry, more than 95% of accidents are caused by miners’ unsafe behaviors [4]. The concept of unsafe behaviors was first introduced in 1931 by Heinrich, who believed that unsafe behaviors of people and objects result from human shortcomings [5]. As front-line workers, coal miners are direct victims of safety accidents, but their dangerous behaviors are also significant causes of accidents.

Previous research and experience showed that most accidents can be prevented [6]. With the development of intelligent mines, which is a new type of safe, efficient, and ecologically green operation based on the achievements of mine automation, digitalization, and informatization, the Internet of Things, cloud computing, artificial intelligence, machine equipment, etc. are integrated with modern mine development technology to form a complete intelligent system for mine interconnection. The working conditions of miners and the safe production environment of coal mines have been greatly improved [7]. The number of fatal coal mine accidents and the death rate of coal per million tons decreased from 5670 and 5.28 in 2001 to 228 and 0.059 in 2020, respectively [8]. However, the situation of coal mine production safety is still not optimistic, and intelligent mines have put forward higher requirements for miners and brought new challenges for coal mine safety management. In intelligent mines, there are many changes in the production safety environment and operational equipment, and miners and safety managers need to improve safety practices. To adapt to the coal enterprises’ intellectual development, it is necessary to develop and foster the creative problem-solving skills of miners [9], and miners’ learning and application of intelligent devices in intelligent mines should solve the creative problem, an inexhaustible motivation for the realization of their self-worth, organizational innovation, and continuous development [10]. Besides, miners need to accumulate appropriate knowledge about intelligent equipment and management, and they face more significant psychological pressure due to the pursuit of self-worth, consideration of family expectations, and extensive creative cognitive and problem-solving processes [11]. It can be seen that intelligent mines place higher demands on miners’ abilities in terms of production processes, knowledge reserves, psychological quality, learning ability, and coal mine safety management. While miners’ unsafe behaviors remain the main cause of coal mine accidents and affect by multiple influences from the individual miner and the external environment, which is different from the traditional process of behavior formation and transmission. To realize increased coal production and reduced accidents in intelligent mines, coal companies and miners face more regulatory requirements. Therefore, it is an urgent problem for safety management changes in the context of intelligent mines to effectively reduce the error rate and injury rate of coal mine workers and to enhance coal mine safety management.

This paper aims to explore the key influencing factors and factors’ hierarchical structure of miners’ unsafe behaviors in intelligent mines, which is a supplement to the theoretical study of miners’ unsafe behaviors and provides a scientific basis for effectively improving the safety management. The overall research framework will provide some references for policy-makers to understand the interrelationship between influencing factors. In our work, (1) we identify 20 influencing factors based on literature research, miners’ interviews, and experts’ recommendations from four dimensions: individual, device, management, and environment, and further, we investigate the relationship and hierarchy of factors; (2) we introduce fuzzy theory to improve the decision-making trial and evaluation laboratory (DEMATEL) method and the maximum mean de-entropy (MMDE) method to exclude unnecessary information in the influence matrix; (3) the interpretive structural modelling (ISM) and MICMAC (Matrix of Cross-Impact Multiplications Applied to Classification) are further introduced, and a novel multi-criteria decision model fuzzy–DEMATEL–MMDE–ISM–MICMAC is developed to understand the interactions and relationships among factors.

This paper consists of five parts: (1) the introduction; (2) the literature review, which contains intelligent mine, factors influencing miners’ unsafe behaviors, multi-criteria decision-making (MCDM) model, and research value and innovation; (3) the methodology, which contains data collection, fuzzy–DEMATEL, MMDE algorithm, and ISM–MICMAC; (4) the results and discussion; (5) the conclusions and remarks.

## 2. Literature Review

### 2.1. Intelligent Mine

**Figure 1:**
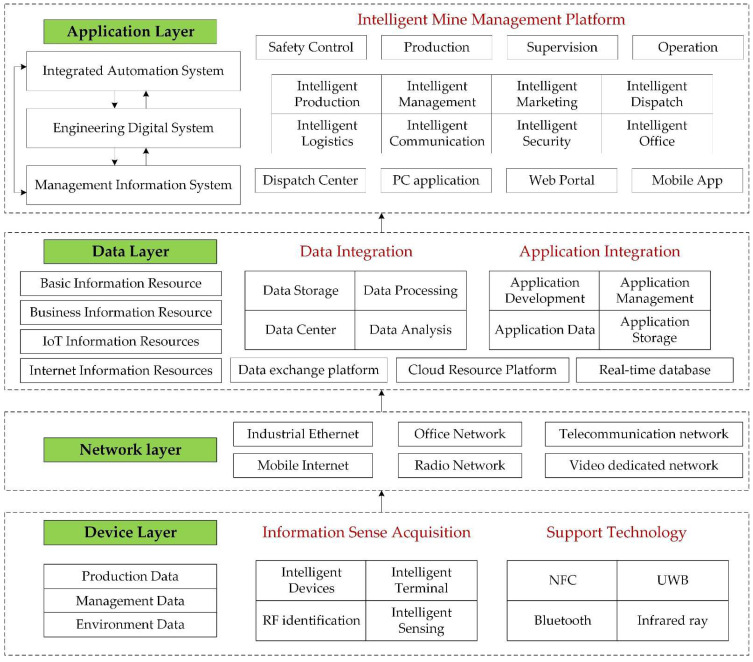
Intelligent mine general system framework in China.

Intelligent mine construction represents the development direction of advanced productivity, and is an important support to achieve high-quality development of coal industry. Intelligent mines are based on the modern wisdom concept, the industrial big data, artificial intelligence, and other deep integration with modern coal development and utilization, and it will form an intelligent system with comprehensive perception, real-time interconnection, independent learning, dynamic prediction and collaborative control, which break the barriers between “human, machine, environment and management” and realize the intelligent operation of the whole process [12]. It is a new type of mine that is safe, efficient and clean, based on the digitalization and informatization of the mine, will be a mine for rapid processing and automatic analysis of production safety and occupational health technology [13]. In China, intelligent mine systems generally include application layer, data layer, network layer, and device layer to provide comprehensive informational control of the whole process of coal mine production safety, occupational health and safety (Figure 1).

With the rapid development of intelligent mines, the intelligent operation of mine production safety processes has been realized [14], which has greatly improved the working conditions of miners and the production safety problems of coal enterprises [7]. However, the safety behavior management of miners will also face new changes and challenges, and the learning and application of intelligent devices by miners in intelligent mines will have to solve creative problems, which requires the development and cultivation of miners’ innovative abilities [9]. The accumulation of corresponding knowledge of intelligent devices and management will face a wide range of innovative cognitive and problem handling processes, leading to greater psychological stress [11]. The process of change in miner safety behavior is different from the traditional behavior formation and propagation process and is subject to multiple influences from the individual miner and the organization’s external environment. Moreover, with the rapid advancement of emerging technologies, today’s intelligent mine production safety has made a qualitative leap from the past, and the dynamic adjustment of production methods requires that the miner safety behavior should also change adaptively [15]. It is very necessary to explore the new changes in miners’ safety behavior and safety management in intelligent mines to further enhance the modern production safety of coal enterprises.

### 2.2. Factors Influencing Miners’ Unsafe Behaviors

Lewin believed that individual characteristics, environmental characteristics influence human behaviors [16]. The formation mechanism of miners’ safety behavior can be analyzed from internal and external causes, from which internal causes emphasize people’s own quality, which mainly includes physiological, psychological and ability aspects, while the external causes highlight the interference of the external environment on people, and the main reasons are organization, management, physical environment, living environment, safety culture, etc. [17]. Moreover, work demands such as work environment, work stress, work intensity, risks and hazards, site safety management, work–family conflict, and work resources such as safety culture, safety climate, education and training, leadership support, communication feedback, and life well-being act together on miners’ safety behavior through attrition processes and motivational processes [18,19]. Based on the above findings, combining the accident causation theory of modern system theory [20], we further consider management and equipment characteristics and investigates the influencing factors of miners’ unsafe behaviors in intelligent mines from four dimensions: individual factors, device factors, management factors, and environmental factors.

#### 2.2.1. Individual Factors

Individual unsafe behaviors are the main cause of safety accidents. Askaripoor’s findings from a questionnaire survey of 115 workers showed a significant correlation between unsafe behavior and safety psychology [21]. Siu argued that workers’ personality characteristics influence accident propensity [22]. Yu et al. studied coal miners’ dangerous behaviors influencing accidents [23]. In addition, Gracia and Martínez-Córcoles found that role stress can lead to workplace risky behaviors and trigger safety accidents among employees [24]. Accident investigations have shown that worker states are essential factors contributing to unsafe behaviors [25]. Nasab et al. first discussed the evolution of unsafe behaviors in workers’ operating processes through factors such as workers’ attitudes, job involvement, job satisfaction, and organizational commitment [26]. Therefore, miners’ personal traits and physical conditions still affect their unsafe behaviors.

Intelligent mines have put forward higher requirements for miners’ ability. Marifran argued that the low safety literacy of individual miners, including lack of safety knowledge, poor safety awareness and poor safety skills, and consequent inability to effectively identify hazards, can easily lead to misconduct [27]. Moreover, Ouellette et al. stated that past behavioral experiences of individuals in complex environments could affect individual behaviors [28]. Liang verified, through structural equations, that accident experience significantly affects miners’ intentions to behave unsafely [29]. Through case studies, Paul and Maiti evaluated the importance of behavioral factors in accidents and injuries in coal mines. They showed how workers might undertake more risky, unsafe behaviors because of unhealthy emotions and job dissatisfaction [30]. Additionally, individual safety perceptions influence unsafe behaviors [31,32], in which the level of knowledge affects the risk perception [33]. Johnson and Hall found that personal subjective norms and perceived behavioral control can moderate the relationship between attitudes and intentions to behave safely [34]. Thus, miners’ knowledge accumulation ability, self-efficacy, and risk perception affect their unsafe behavior, which complements the work of Wang et al. [35], and reflects the new requirements and challenges facing miners in intelligent mines.

#### 2.2.2. Device Factors

There is a meaningful relationship between operating equipment and miners’ unsafe behaviors. Zhang et al. indicated that the complex operating environment of coal mines poses more risks to miners [36]. Smart devices improve reliability under challenging conditions and provide an improved operating safety environment for miners such that miners need only monitor and operate from the control center [37]. Based on Internet of Things (IoT) systems and smart devices for efficient production in smart coal mines, electromechanical and monitoring systems have been added to consider the impact of equipment levels on miner safety [38,39]. Additionally, the unsafe status of the equipment, i.e., the relevant personnel failing to test and maintain the equipment in accordance with national regulations, which would eventually result in miners’ unsafe behavior [40]. Furthermore, the high mobility of coal mine production operations, the ever-changing operating environment, and the frequent movement of operating equipment greatly reduce the reliability of systems and equipment, and must rely on the correct disposal behavior of miners to compensate for deficiencies [41]. Moreover, Krause showed that improving technology cannot lead to any stabilization of mine accident rates, making it necessary to consider human factors to reduce accidents [42]: miners’ use of intelligent devices and the degree of matching should be considered comprehensively, thus reducing the probability of accidents. Intelligent machines affect miners’ operations. Ashis et al. studied 516 underground workers: their use of work tools and work posture can cause physical injuries to employees. Additionally, the number of work tasks significantly influences the incidence of injuries [43]. Based on the above findings, intelligent comprehensive mining equipment level, intelligent device level, intelligent device security status, man–machine compatibility and operating intensity of intelligent equipment affect miners’ unsafe behaviors, which all reflect the new changes brought by the background of intelligent mines.

#### 2.2.3. Management Factors

Manogaran et al. explored the changing trends of human factors which lead to coal mining accidents [44]. Loiselle et al. analyzed mining accidents, and found that employees shared the same perception of management’s attention to safety [45]. Li et al. concluded, from a questionnaire survey of 200 employees, that management charismatic leadership style significantly influences miners’ unsafe behavior; safety-related attitude is a mediating variable that also affects miners’ unsafe behaviors [46]. Furthermore, Burcak argued that the coal mining companies have a conflict between maximizing profits and improving miners’ safety [47]. Thus, the importance of leadership, as well as the level of safety input, is still one of the influencing factors of miners’ unsafe behaviors in intelligent mines.

In the modern mine production safety system, it is difficult to achieve the desired effect using rewards and punishments as an essential tool of current risk management [48]: it is necessary to focus on the improvement of the safety system. In addition, Cao et al. qualitatively modeled the evolutionary patterns of miners’ unsafe behaviors and found that external interventions can inhibit the spread of unsafe behaviors [49]. Moreover, Kumar found that it is difficult for coal mining companies to respond effectively to emergencies without a sound risk pre-control management model [50]. Therefore, standardization of safety systems, dynamic supervision intensity and emergency management capacity affect miners’ unsafe behaviors, which also complements the work of Wang et al. [35].

#### 2.2.4. Environmental Factors

Operating environment comfort is clearly an important effect of miners’ unsafe behaviors. Underground mining is one of the main parts of coal mining operations. The interplay of harsh microclimatic conditions, narrow operating spaces, and heavy workloads leads to underground mining accidents [35]. Cui et al. used structural equation modeling to reveal the causal association between hazardous environment, safety climate, and personal safety behaviors. They found that employees’ perception of a dangerous environment significantly affects employees’ safety behaviors [51]. Additionally, noise from equipment can affect both the human body and the mind [52], thus affecting behaviors. Maiti found that the features of miners’ workplace have a significant impact on the occurrence of accidents, such as under noisy workplaces, miners cannot concentrate effectively and are prone to unsafe behaviors [53]. Furthermore, Zhang et al. discussed the state of coal miners in different production environments and argued the detection of the coal mine environment has been a crucial part of coal mine production [54]. Additionally, Tuna et al. discovered that the corporate safety climate and the importance of the organization to employees were negatively correlated with employees’ unsafe behaviors [55]. Samuel found that organizational culture has an important influence on the transmission of unsafe behaviors, and the work environment impacts the transmission of unsafe behaviors [56]. Fang et al. and Siu et al. focused on the effects of safety climate on the emergence and development of individual unsafe behaviors [57,58]. Casey and Krauss found that joint staff safety support and communication showed effective relationships with safety [59]. Moreover, individual risk perceptions are profoundly influenced by the work safety atmosphere [60].

In addition, Harvey suggested that the government’s failure to develop an effective legal system would lead to the blurring of safety legal boundaries, and then coal mining companies would belittle the importance of miner safety, which in turn would increase the risks of miners’ work [61]. From the perspective of family atmosphere, Wang et al. conducted an empirical study and found that family environment and work stress are closely related to insecure behaviors [62]. Thus, the government regulation and family safety expectations affect miners’ unsafe behaviors, which is different from previous studies and reflects the fact that with the development of smart mines. Miners are constantly adapting to changes in their environment and focusing more on their own satisfaction.

### 2.3. Multi-Criteria Decision-Making (MCDM) Model

Multi-criteria decision-making (MCDM) models rank feasible options in order of best or worst by comparing them using a set of conflicting criteria [63]. One of these criteria, DEMATEL, is popular in many areas, including security management [64]. This method investigates the relationship between causal and central factors [65], and is used to list variables from those related to the problem [66]. Wang et al. used a system hierarchical system to research factors influencing coal production safety and likewise performed DEMATEL analysis on the secondary indicators in this system [35]. In many cases, DEMATEL judgments are often given specific values that have insufficient ambiguousness to reflect the real world [67]. Subjective judgments exist for expert evaluations in DEMATEL. Human preferences are hard to evaluate with accurate numbers. Fuzzy logic deals with ambiguity and imprecision [68,69], handling the weakness of the decision cycle [70]. Therefore, fuzzy logic is needed to improve the DEMATEL method to make more appropriate decisions in an ambiguous environment. Fuzzy DEMATEL is used to deal with the bias and ambiguity inherent in human judgment [71] and the problem of group decision making under vague conditions [72]. Ahmadi et al. mapped the fuzzy DEMATEL output into Bayesian networks. Prior indicators were devised for risk-influencing factors. Their content validity, usefulness, and importance were assessed using the fuzzy logic method [73].

The primary role of the DEMATEL is to quantitatively estimate the importance degree, thus further highlighting the strength of factors, but it cannot cascade all influencing factors. The integration of ISM and DEMATEL models is to understand the relationship of influencing factors better. The ISM and DEMATEL methods are improved using fuzzy theory to clarify the relationship between factors within the system [35,74]. The relationship between factors can be investigated through combination of the fuzzy–ISM–DEMATEL approach [75]. Guangli et al. used DEMATEL and ISM methods to study miners’ unsafe emotions. Multiple influencing factors can adversely affect miners’ psychology, which breeds destructive emotions and affects miners’ safe production behaviors [76]. Wang et al. used DEMATEL–ISM to determine the security factors in coal mines [35].

The process of DEMATEL combined with the ISM method needs a suitable threshold value to obtain enough information for in-depth analysis. Most thresholds in existing studies are determined jointly by experts [77,78], which is limited by subjective judgment. Some scholars use the mean value method [79,80] to determine the threshold value; during the process, nearly half of relationships of influencing factors are artificially removed, which prevents determination of accurate thresholds. Some other scholars used the method of statistical distribution to determine the thresholds [81], which essentially assumed that the data were normally distributed (and may not be consistent with reality). The maximum mean de-entropy (MMDE) method [82] was applied to reduce the amount of information and determine thresholds to integrate DEMATEL and MMDE. It aims to analyze problems effectively and provide recommendations. Lee and Lin integrated the DEMATEL and MMDE methods; they analyzed the financial ratios of shipping companies [83]. Behera and Mukherjee explored the critical influences on selecting supply chain coordination options with the DEMATEL and MMDE integrated approach [84]. Singh and Bhanot used MMDE to determine the thresholds of the integrated approach, analyzing barriers to IoT implementation in manufacturing by integrating multiple decision methods [85]. The Matrix of Cross-Impact Multiplications Applied to Classification (MICMAC) approach determines the interaction between factors through the reachable matrix of ISM. Shanker and Barveb explored supply chain sustainability using an integrated fuzzy–ISM–MICMAC–DEMATEL approach [86]. Shakeri and Khalilzadeh integrated the fuzzy–DEMATEL–ISM–MICMAC approach to study project communication factors [87], but none of them used the MMDE method to determine objective thresholds.

### 2.4. Research Innovation

At present, there are many studies on miners’ unsafe behaviors, but research into miners’ unsafe behaviors and their influencing factors in the context of intelligent mines remains sparse, and there is little research on the mechanism of mutual influence and hierarchical relationship among various factors. In many fields, the DEMATEL, ISM, MICMAC, and other multi-criteria decision-making methods are used, which laid the theoretical foundation of this study. However, fewer scholars consider the integrated compensation of multiple objective deficiencies in the integration process of decision methods and few use the MMDE method to determine the unique objective threshold in the integration process of DEMATEL–ISM–MICMAC. Furthermore, few scholars have integrated this method into the work of miners’ unsafe behaviors in intelligent mine conditions. It is important to explore the key influencing factors of miners’ unsafe behaviors in intelligent mines.

Therefore, we introduce the MCDM into the analysis of miners’ unsafe behaviors under intelligent mines and study the relationship and hierarchy of 20 factors. We use the fuzzy theory to improve the DEMATEL method and the more objective the converting fuzzy data into crips scores (CFCS) method for defuzzification, which aimed to determine the causal relationships of the influencing factors. In particular, we introduce the MMDE method to determine the unique threshold scientifically. Furthermore, we integrate the ISM method and the MICMAC method. A new multi-criteria decision-making model fuzzy–DEMATEL–MMDE–ISM–MICMAC is developed, which strive to provide references for preventing accidents and improving safety management in coal mines.

## 3. Methodology

**Figure 2:**
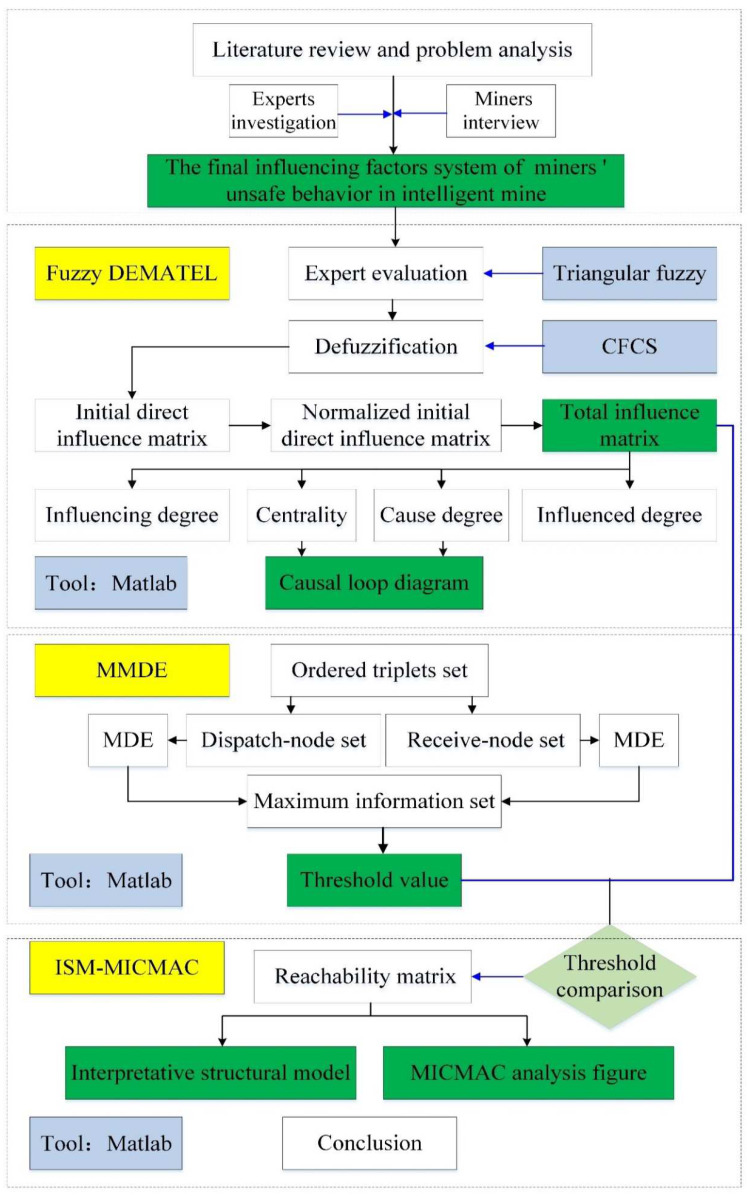
Technical procedure used in the influencing factor analysis.

This paper uses the fuzzy–DEMATEL–MMDE–ISM–MICMAC integrated approach and revealed the interrelationship and hierarchical structure among the factors influencing the miners’ unsafe behaviors in intelligent mines. The technical procedure used through the present study is shown in Figure 2.

### 3.1. Data Collection

**Figure 3:**
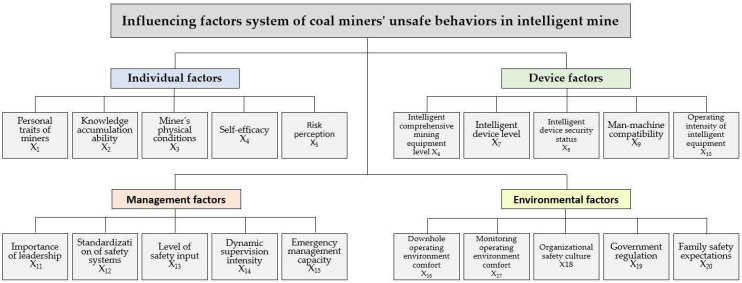
Influencing factors system of coal miners’ unsafe behaviors in intelligent mine.

This study takes Shaanxi Coal Yubei Coal Industry Xiaobaodang Mining Co which it is in Yulin, Shaanxi Province, China as an example. Academics generally agree that group decision-making with five to seven people is the most effective [88]; therefore, one safety manager of the coal mine, four representatives of miners, and two professors engaged in coal mine safety management and system decision-making research were invited. The opinions of the seven experts were used as the data for the decision analysis. First, we conduct a preliminary analysis of the factors influencing the incomplete behavior of miners in intelligent mines combing through relevant literature and accident cases. Based on this, seven experts are invited to revise the index system of factors influencing the miners’ unsafe behaviors in intelligent mines, and discuss the accuracy and independence of the description of influencing factors: 20 influencing factors were finally identified (Figure 3). Next, we invite experts to assess the relationship between two factors using the linguistic operators “No impact (No)”, “Very low impact (VL)”, “Low impact (L)”, “High impact (H)”, and “Very strong impact (VH)”.

In individual factors, personal traits of miners *x*_1_ mean the educational level, behavior habits, safety literacy, and personality traits of miners. Knowledge accumulation ability *x*_2_ represents the knowledge learning and accumulation ability and knowledge skill level of miners, which is a requirement for the ability to efficiently and safely accomplish production operations in intelligent mine. Miners’ physical conditions *x*_3_ include fatigue state, material parameters, biological rhythm, emotion, and mentality. Coal mining companies should fully consider the physical condition of miners to safeguard their occupational health. Self-efficacy *x*_4_ indicates the extent of miners’ self-worth reinforcement from the perspective of the hierarchy of needs theory. Risk perception *x*_5_ refers to safety recognition, risk perception, and emergency decision-making level of cognition.

In device factors, intelligent comprehensive mining equipment level *x*_6_ is the total level of smart-mining equipment instruments such as coal mining equipment, hydraulic frame, transportation equipment, and coal mine coverage. Intelligent device level *x*_7_ represents the comprehensive level of real-time monitoring equipment, data transmission system, sensors, actuating equipment, and coal mine coverage. Intelligent device security status *x*_8_ denotes the health monitoring of equipment, equipment operation, and maintenance. Man–machine compatibility *x*_9_ refers to the level of miners’ equipment operation matching and miners’ proficiency in operating equipment, which is a suitable combination of miners and machines that can efficiently manipulate the devices for safe coal mine production. The operating intensity of intelligent equipment *x*_10_ is the labor intensity of intelligent mining equipment operations and the level of health hazards facing coal mine occupations.

In management factors, the importance of leadership *x*_11_ refers to the complete management concept, adopting a variety of safety management behaviors, and improving the management level. Standardization of safety systems *x*_12_ refers to the standardization of safety management, safety training, incentives, supervision, and other systems, which reflects the need to enhance the standardization of coal mine safety to reduce accidents while ensuring the production of coal mines. Level of safety input *x*_13_ is the degree of safety input to safety management, equipment updating, and organization training. Dynamic supervision intensity *x*_14_ indicates the intensity of safety supervision, information detection, and process management. Emergency management capacity *x*_15_ is the capacity of emergency preparedness, emergency response, emergency disposal, and emergency recovery.

In environmental factors, downhole operating environment comfort *x*_16_ is the suitability of the working environment as affected by environmental conditions such as noise, dust, temperature, humidity, and lighting. Monitoring operating environment comfort *x*_17_ represents the ecological parameter monitoring, equipment status monitoring, continuous monitoring, warning system, etc., used to monitor the operating environment. Organizational safety culture *x*_18_ indicates the organizational setting such as safety awareness, corporate equity, organizational innovation and change, and interpersonal communication. Government regulation *x*_19_ is the intensity of government regulation. According to the hierarchy of needs theory, family safety expectations *x*_20_ represent the safety expectations of individuals and families of miners.

### 3.2. Fuzzy–DEMATEL

Fontela and Gabus first proposed a decision-making trial and evaluation laboratory [65], which can use expert experience and knowledge to identify factors within complex networks and analysis [89]. It is also based on matrix tools and graph theory to clarify the causal relationships and importance ranking of factors [90]. The DEMATEL method is based on expert experience and knowledge, which is subjective and affects the research results; therefore, it can use a combination of Fuzzy Set Theory and DEMATEL. It incorporates fuzzy triangular numbers into the traditional DEMATEL method. The direct influence matrix is fuzzified by converting the semantic assessment of the experts into the corresponding triangular fuzzy numbers [91]. The steps are as follows:

**Step 1:** The system of factors influencing miners’ unsafe behavior in intelligent mines is constructed and set to *x*_1_, *x*_2_, *x*_3_, …, *x_n_*.

**Step 2:** Inviting experts to assess the relationship between the two factors using the linguistic operators “No impact (No)”, “Very low impact (VL)”, “Low impact (L)”, “High impact (H)”, and “Very strong impact (VH)”. Based on the settings of the experts’ linguistic variables by Wang and Chang [92], Table 1 shows the fuzzy linguistic scales [71,93]. The original evaluations were transformed into $w_{ij}^{k}=(l_{ij}^{k},m_{ij}^{k},r_{ij}^{k})$, representing the fact that the *k*^th^ expert believes factor *i* influences factor *j*, *l* is the left-hand side value that is the conservative value, *m* is the median value closest to the actual value, *r* is the right-hand side value that is the optimistic value, and *l* ≤ *m* ≤ *r*.

**Step3:** Using the CFCS to defuzzify initial values of expert scores [94]: this leads to the *n*-order direct influence matrix *D*. It includes four links [71]:

(1) Standardizing the triangular fuzzy number

(1) $$xl_{ij}^{k}=\left(l_{ij}^{k}-\min l_{ij}^{k}\right)/\Delta_{\min}^{\max}$$

(2) $$xm_{ij}^{k}=\left(m_{ij}^{k}-\min l_{ij}^{k}\right)/\Delta_{\min}^{\max}$$

(3) $$xr_{ij}^{k}=\left(r_{ij}^{k}-\min l_{ij}^{k}\right)/\Delta_{\min}^{\max}$$

$$\Delta_{\min}^{\max}=\max r_{ij}^{k}-\min l_{ij}^{k}$$

(2) Standardizing the left value and the right value

(4) $$xls_{ij}^{k}=xm_{ij}^{k}/\left(1+xm_{ij}^{k}-xl_{ij}^{k}\right)$$

(5) $$xrs_{ij}^{k}=xr_{ij}^{k}/\left(1+xr_{ij}^{k}-xm_{ij}^{k}\right)$$

(3) Obtaining the clear value after deblurring

(6) $$x_{ij}^{k}=\left[xls_{ij}^{k}\left(1-x{\mathrm{ls}}_{ij}^{k}\right)+x{\mathrm{rs}}_{ij}^{k}x{\mathrm{rs}}_{ij}^{k}\right]/\left[1-x{\mathrm{ls}}_{ij}^{k}+x{\mathrm{rs}}_{ij}^{k}\right]$$

(7) $$z_{ij}^{k}=\min l_{ij}^{k}+x_{ij}^{k}\Delta_{\min}^{\max}$$

(4) Calculating the average clarity value

(8) $$z_{ij}=\frac{1}{k}\left(z_{ij}^{1}+z_{ij}^{2}+\cdots +z_{ij}^{k}\right)$$

**Step 4:** Calculating the standardized direct impact matrix *N.*

(9) $$N=\frac{D}{\max\left[\max\left(\sum_{j=1}^{n}d_{ij}\right),\max\left(\sum_{i=1}^{n}d_{ij}\right)\right]}$$

**Step 5:** The integrated impact matrix represents the direct and indirect effects of the system factors’ combined effect. After successive self-multiplication of the canonical influence matrix, all matrix values converge to zero ($\underset{k\to \infty }{\lim}N^{k}=0$). Therefore, the integrated impact matrix *T* is obtained according to the following equation. *I* is an *n* × *n* unit matrix.

(10) $$T=(N+N^{2}+\cdots +N^{k})=\sum_{k=1}^{\infty }N^{k}=N(I-N{)}^{-1}$$

**Step 6:** Calculating the degree of influence of each element, which indicates the degree of influence of an element in each row on other elements. It is denoted by *D_i_*. Calculating the degree to which it is influenced, which indicates the degree of influence of an element in each column on other elements (denoted by *R_i_*). Calculating the centrality, to indicate the central position and importance of the factor. The degree of centrality is the sum of *D_i_* and *R_i_*. The difference between *D_i_* and *R_i_* is the extent of the causality. If the causality is greater than 0, it is the cause element. Conversely, it is called the resulting factor. The formula is as follows:

(11) $$D_{i}=\overset{n}{\underset{j=1}{\sum }}x_{ij},(i=1,2,\cdots ,n)$$

(12) $$R_{i}=\overset{n}{\underset{j=1}{\sum }}x_{ij},(i=1,2,\cdots ,n)$$

**Step 7:** Drawing the causality diagram.

### 3.3. MMDE Algorithm

Before integrating the DEMATEL and ISM methods, a suitable threshold has to be determined to supplement the information and basis for decision-making judgments. Thresholds are determined mainly by expert evaluation, mean value method, distribution method, etc. Expert evaluation entails subjective judgment, the mean value method does not accurately consider the influence relationship of nearly half of the factors, and the distribution method may not be consistent with the actual situation; therefore, the MMDE algorithm is introduced here. It is used to obtain an objective and accurate threshold [82]. The concept of entropy is applied to information theory and unnecessary information is excluded from the influence matrix. The MMDE algorithm eliminates the need for experts and provides accurate and objective unique thresholds, which are calculated using the following steps [95,96]:

**Step 1:** Converting the total relationship matrix *T* into an ordered set, which is {*t*_11_, *t*_12_, …, *t*_21_, *t*_22_, …, *t_nn_*}. Subsequently, sorting all elements of an ordered set by the size and passing them into the set (*t_ij_*, *x_i_*, *x_j_*).

**Step 2:** Constructing the set of scheduling nodes (*T^Di^*) and the set of receiving nodes (*T^Re^*). Extracting the last two elements of (*t_ij_*, *x_i_*, *x_j_*) to obtain the ordered set of scheduling nodes (*T^Di^*) and the set of receiving nodes (*T^Re^*). The definition is as follows:

(13) $$T^{D_{i}}=\left\{x_{i}\right\}=\left\{x_{1},x_{2},\ldots ,x_{n\times n}\right\}$$

(14) $$T^{R_{e}}=\left\{x_{j}\right\}=\left\{x_{1},x_{2},\ldots ,x_{n\times n}\right\}$$

**Step 3:** Extracting the first t elements of *T^Di^* and obtain a set *T^Di^_t_*. Calculating the probability of components, and then the mean de-entropy value (MDE). First, *t* is raised from 1 to *C*(*T^Di^*), each increment is 1 and *T^Re^* is processed in the same way. The equation is as follows:

(15) $$H_{t}^{D_{i}}=H\left[\frac{1}{N\left(T^{D_{i}}\right)},\frac{1}{N\left(T^{D_{i}}\right)},\ldots ,\frac{1}{N\left(T^{D_{i}}\right)}\right]-H\left[\frac{k_{1}}{C\left(T^{D_{i}}\right)},\frac{k_{2}}{C\left(T^{D_{i}}\right)},\ldots ,\frac{k_{t}}{C\left(T^{D_{i}}\right)}\right]$$

(16) $$H_{t}^{R_{e}}=H\left[\frac{1}{N\left(T^{R_{e}}\right)},\frac{1}{N\left(T^{R_{e}}\right)},\ldots ,\frac{1}{N\left(T^{R_{e}}\right)}\right]-H\left[\frac{k_{1}}{C\left(T^{R_{e}}\right)},\frac{k_{2}}{C\left(T^{R_{e}}\right)},\ldots ,\frac{k_{t}}{C\left(T^{R_{e}}\right)}\right]$$

(17) $$MDE_{t}^{D_{i}}=\frac{H_{t}^{D_{i}}}{N\left(T_{t}^{D_{i}}\right)}$$

(18) $$MDE_{t}^{R_{e}}=\frac{H_{t}^{R_{e}}}{N\left(T_{t}^{R_{e}}\right)}$$

**Step 4:** Determining the maximum value and all elements before the maximum value at the position, and deleting duplicate elements.

(19) $$T_{\max}^{D_{i}}=\max\left(MDE_{t}^{D_{i}}\right)=\left\{x_{1},x_{2},\ldots ,x_{t}^{\max}\right\}$$

(20) $$T_{\max}^{R_{e}}=\max\left(MDE_{t}^{R_{e}}\right)=\left\{x_{1},x_{2},\ldots ,x_{t}^{\max}\right\}$$

**Step 5:** Identifying the threshold. The threshold is the minimum value in *T^Th^*.

(21) $$T^{Th}=\left\{t_{ij},T_{\max}^{D_{i}}\left(x_{i}\right),T_{\max}^{R_{e}}\left(x_{j}\right)\right\}$$

### 3.4. ISM–MICMAC

The DEMATEL model is used to determine the causal relationship between the influencing factors; however, it cannot accurately delineate the hierarchy of influencing factors in the index system [97,98]. ISM decomposes a complex system into several subsystem elements, which eventually constitute a multilevel recursive structural model for analyzing the hierarchical structure among factors [99]. Combining the two can clearly show the relationships within the system [35,74]. Based on the combination of DEMATEL, then the integration of ISM and MICMAC [86,87], the system elements are further classified. It clarifies the role of each factor in the system and the interrelationship between the factors. The methodological steps are as follows:

**Step 1:** Calculating the initial reachability matrix. The threshold determined by MMDE and the combined influence matrix *T* of DEMATEL are applied, excluding the continuous affectivity and considering the influence of factors on themselves, and the final reachability matrix *K* is determined.

(22) $$H_{ij}=\left\{ \begin{array}{l} 1,t_{ij}\geq \lambda \\ 0,t_{ij}<\lambda \end{array}\right.(i,j=1,2,\ldots ,n),H={\left[h_{ij}\right]}_{n\times n}$$

**Step 2:** Calculating antecedent set $A\left(s_{i}\right)$ and reachable set $R\left(s_{i}\right)$.

(23) $$R\left(s_{i}\right)=\left\{s_{j}\in S|k_{ij}=1\right\}$$

(24) $$A\left(s_{i}\right)=\left\{s_{j}\in S|k_{ji}=1\right\}$$

(25) $$B\left(s_{i}\right)=\left\{s_{i}\in S|R\left(s_{i}\right)\cap A\left(s_{i}\right)=A\left(s_{i}\right)\right\}$$

where $B\left(s_{i}\right)$ is the top-level factor set.

**Step 3:** Mapping the explanatory structure model.

**Step 4:** The system elements are classified using the MICMAC. Driving force is the sum of the values in the rows of the final reachable matrix for that element, indicating the extent to which it is influenced by other metrics. Dependency, the degree to which it is influenced by other indicators, is the sum of the values of the columns from the final reachable matrix where the element is located.

**Step 5:** Drawing the MICMAC analysis diagram. The dependency values and driving force values for each factor are calculated. Then, a right-angle coordinate system with horizontal coordinates representing dependencies and vertical coordinates representing drivers is constructed.

## 4. Results and Discussion

### 4.1. Results Analysis: Fuzzy-DEMATEL

Using the expert scoring method, seven experts compared the influence of *x_i_* on *x_j_*. They judged the relationship between the two factors based on the criteria in Table 1. Moreover, the diagonal line of the direct influence matrix is denoted as “No” because the factor does not influence itself and the direct influence matrix is determined. The scoring data provided by one of the professors and the miners from China Shaanxi Coal Yubei Coal Industry Xiaobaodang Mining Co which is in Yulin, Shaanxi Province, China are shown in Appendix A and Appendix B, respectively.

**Figure 4:**
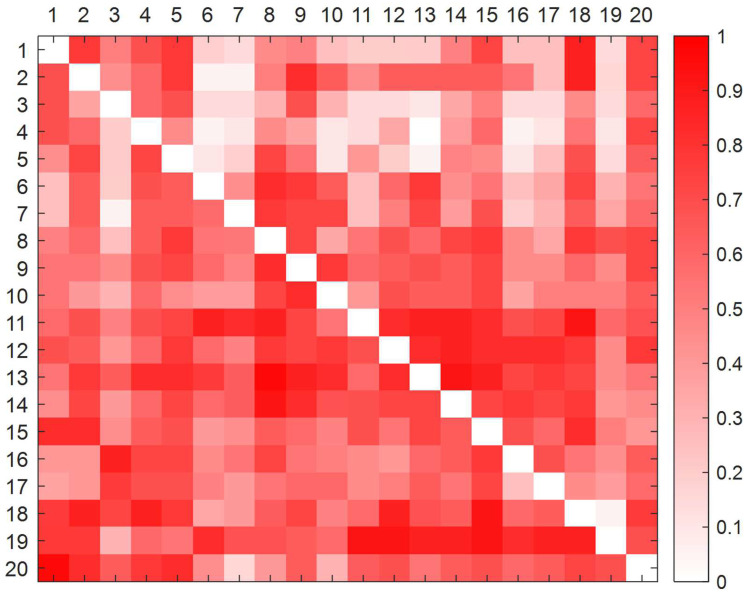
Defuzzified direct influence matrix plot.

**Figure 5:**
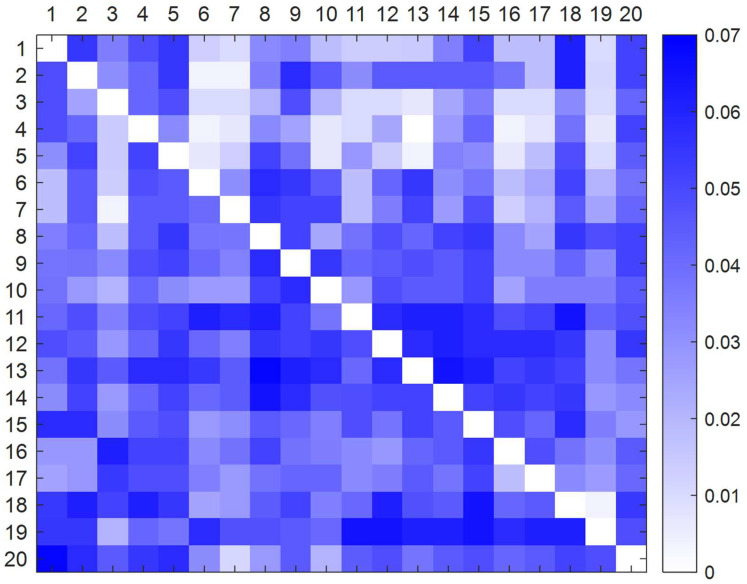
Normalized direct influence matrix plot.

The deblurred direct impact matrix is calculated from formulas (1) to (8) (Appendix C). The standardized direct influence matrix is then determined from formula (9). The deblurred direct influence matrix is plotted with MATLAB™ software (Figure 4) and the standardized direct influence matrix is drawn (Figure 5). In order to understand the direct influence relationship between factors more intuitively: the deeper the influence of the factors in that row on the factors in that column, the darker the color in the connected graph.

**Figure 6:**
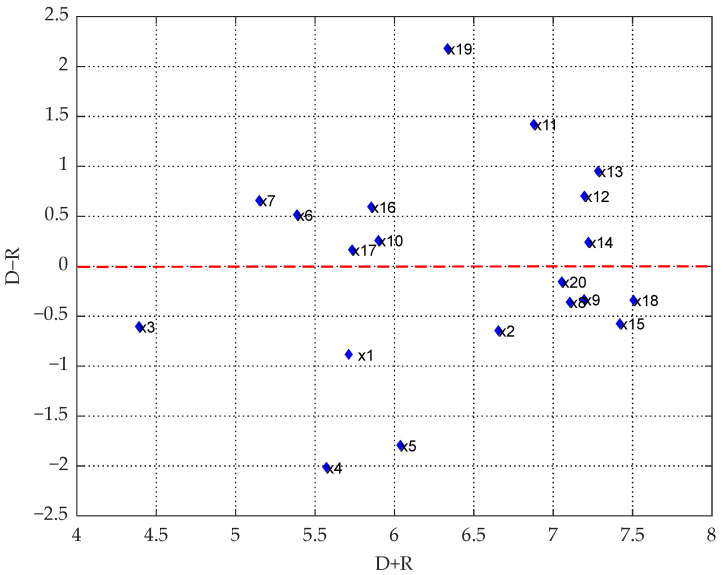
Causal diagram for factors influencing unsafe behaviors.

Based on formula (10), the matrix calculation was performed using MATLAB™. This allows us to determine the integrated influence matrix (Table 2). Specific values of each influencing factor are calculated based on formulas (11) to (14), as shown in Table 3. MATLAB™ software is used to plot the causality diagram (Figure 6).

From Table 3, it can be seen that the influence degree of government regulation (*x*_19_), leadership attention (*x*_11_), safety input level (*x*_13_), safety system standardization (*x*_12_), and dynamic supervision intensity (*x*_14_) are the five factors that exert the most significant influence on the others. Among them, the strength of government regulation most significantly influences other factors, which belong to environmental factors. The other four factors are all management factors, showing that the management of miners under intelligent mines plays a crucial part in controlling unsafe behaviors. The government increases in supervision, and leadership pay more attention to improving safety investments such as intelligent equipment, dynamic supervision, and staff training, so can enhance the standardized management of safety systems, which influences other factors, thus effectively controlling the process of safe coal mine operation.

The centrality (*D* + *R*) reflects the importance of the factors. From Table 3 and Figure 6, *x*_18_ (organizational safety culture) is shown to have the highest centrality. It needs to improve the organizational safety climate and create an excellent organizational safety culture. In addition, among the individual factors, *x*_2_ (knowledge accumulation ability) shows the highest centrality, indicating that the knowledge accumulation ability of miners is essential. It is necessary to manage miners scientifically and rationally in accordance with their characteristics and give full play to their comprehensive ability to control coal mine safety effectively. Among the device factors, *x*_9_ (man–machine matching) has the highest centrality, indicating that miners have to effectively use intelligent equipment. Among the management factors, *x*_15_ (emergency management capability) exhibits the highest centrality, so one should focus on the quality improvement of the whole process of emergency management. It is essential to improve the level of unsafe accident prevention and emergency management. Among the environmental factors, *x*_18_ (organizational safety culture) shows the highest centrality, so maintenance and protection of the organizational safety culture are required.

The degree of causality (*D* − *R*) indicator is positive or negative, and works in opposite directions: if it is positive, it is a causal factor, so it needs the positive control of such influencing factors. If it is negative, it is a resulting factor. These factors are influenced by other factors and thus influence unsafe behaviors. From Table 3 and Figure 6, *x*_19_ (government regulation) has the highest causality. It affects other factors, proving that government regulation plays an essential role in safety management. Factor *x*_4_ (self-efficacy) displays the smallest causality, which is negative, meaning that it is most susceptible, which means that miners’ self-worth enhancement is most sensitive to various other factors. It is essential to focus on the self-efficacy enhancement of miners. Through comprehensive control of various influencing factors, miners’ self-worth perception and organizational sense of belonging can be improved.

### 4.2. Results Analysis of MMDE

The threshold value of integrated DEMATEL- ISM was calculated by using formulas (13) to (21), and the calculation process and results of MMDE are listed in Table 4. The final threshold value was determined to be 0.2463.

### 4.3. Results Analysis: ISM–MICMAC

**Figure 7:**
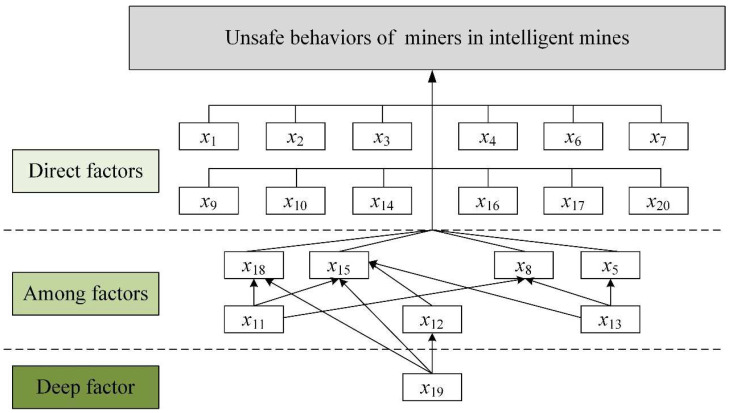
ISM model of miners’ unsafe behaviors in intelligent mines.

Based on the total influence impact matrix and threshold, the initial reachability matrix is obtained from formula (22) and the final reachability matrix is obtained (Appendix D). Considering the influences of factors on themselves and the transferability between factor influences, from formulas (23) and (24), the antecedent and reachable sets are established, and the hierarchy of factors is determined (Appendix E). Based thereon, the explanatory structure model diagram is drawn (Figure 7).

From Figure 7, the deep factor is government regulation (*x*_19_), which has the most pronounced effect. Government regulation affects the normality of the safety system of an organization, the ability of emergency management, and the safety culture of the organization, which in turn affects other factors. It is the deep cause of miners’ unsafe behaviors in intelligent mines. The intermediate factors include leadership attention (*x*_11_), safety system standardization (*x*_12_), and safety input level (*x*_13_), which play a part in the structure of the model, are influenced by the deep factors, and also influence other factors. Other factors may be classified as factors directly affecting the unsafe behaviors of employees in intelligent mine.

**Figure 8:**
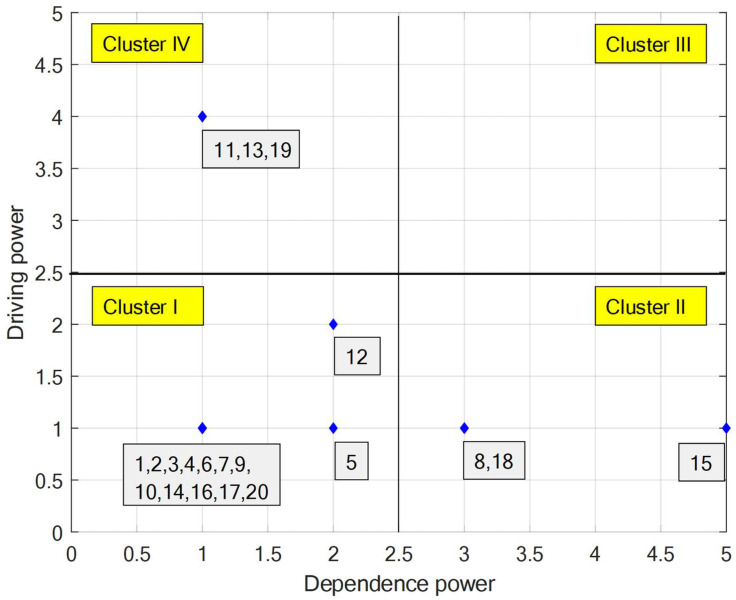
MICMAC analysis of influencing factors of unsafe behaviors.

The driving force value and dependency value of each factor are calculated by the final reachable matrix (Appendix F). Positioning the 20 influencing factors in the coordinate system, the results of MICMAC analysis are obtained (Figure 8).

From Figure 8, the autonomy factor Cluster I contains miners’ personal traits (*x*_1_), knowledge accumulation ability (*x*_2_), miners’ physical condition (*x*_3_), self-efficacy (*x*_4_), risk perception ability (*x*_5_), level of intelligent integrated mining equipment (*x*_6_), level of intelligent sensing equipment (*x*_7_), human–machine matching (*x*_9_), intensity of intelligent equipment operation (*x*_10_), safety system normality (*x*_12_), dynamic supervision intensity (*x*_14_), the comfort of the underground operating environment (*x*_16_), the comfort of the monitored operational environment (*x*_17_), and home safety expectation (*x*_20_). These factors are less driven and dependent but have a direct influence.

Dependency factors in Cluster II contain the security status of intelligent devices (*x*_8_), emergency management capabilities (*x*_15_), and organizational security culture climate (*x*_18_). These factors are weak drivers, while their dependency is higher than other factors, indicating that they are more susceptible to influences of other factors. Management has to pay attention to controlling these essential factors to avoid interference of other influencing factors to the safety status of intelligent devices, emergency management capabilities, and organizational safety culture. These factors may lead to miners’ unsafe behaviors.

The system does not store influencing factors in the linkage factor set (Cluster III).

The driving factors (Cluster IV) contain leadership attention (*x*_11_), the level of security investment (*x*_13_), and government regulation (*x*_19_). It is a set of independent factors with higher drive and lower dependence. These factors are less significantly influenced by other factors but are deep core factors influencing other factors, which need to be controlled more carefully. Leaders should improve the level of safety investment, including updating intelligent equipment and organizing training, and increasing government supervision intensity to control unsafe behaviors more effectively. These influencing factors will lead to the top of the ISM hierarchy and should be prioritized.

## 5. Conclusions and Remarks

Coal mine safety management concerns people’s lives and society’s stability development: at present, it is the key to change for coal enterprises to manage miners in intelligent mines. Moreover, coal mine safety management and miners’ work in intelligent mines face new changes and higher requirements. To guarantee the production of coal while also taking full account of the safety and occupational health, coal mining companies and miners face more new challenges. Therefore, this article analyzed the influencing factors of miners’ unsafe behaviors in intelligent mines. Our work can be seen as an extension and complement to the work of Wang et al. [35], where we studied new changes in the factors on the intelligent mine context. We identified 20 influencing factors from four dimensions: individual, device, management, and environment. Fuzzy set theory is introduced to improve the DEMATEL method, and the CFCS is used for defuzzification. This allows us to determine the causal relationship between each influencing factor. It also reveals the weak and robust relationship between influencing factors and the influencing mechanism. The MMDE method is introduced to determine the accurate threshold objectively. The ISM method is then used to delineate the hierarchy of factors. Finally, the MICMAC is used to determine the interdependence between the factors of miners’ unsafe behaviors. The overall research framework will provide some references for policy-makers to understand the interrelationship between influencing factors and prevent accidents and occupational disease hazards. The main conclusions were drawn as follows:

(1) Among the individual factors, it is necessary to focus on the direct influence of human factors on accidents and highlight the improvement of miners’ self-efficacy and knowledge accumulation ability. Among all the influencing factors, self-efficacy is most easily influenced by other factors. Attention should be paid to the enhancement of miners’ sense of self-worth and organizational belonging. Additionally, it needs to notice the influences of other influencing factors on miners’ sense of self-efficacy. Knowledge accumulation ability shows the highest centrality among the individual factors. This should be combined with the personal characteristics of miners to improve rational management thereof. Coal mining enterprises should improve individual working environments and guiding coal miners to create career plans. Further, they should establish a sound safety responsibility system and dynamic reward and punishment mechanism, which effectively guarantee the safety of miners’ lives and property and personal development requirements.

(2) Among the device factors, the degree of man–machine compatibility has the highest centrality. It is necessary to improve use efficiency of intelligent equipment, and strengthen miners’ technical training and safety control. Enterprises should use mentor-expert training activities to effectively improve the human–machine match with individual worker characteristics. The intelligent equipment safety status shows a high degree of dependence and is easily affected by other factors. Technological innovation and solution modification design should be given full consideration to deal with the practical problems faced by the integrated mining work. Management effectively prevents the interference of other influencing factors to control miners’ unsafe behaviors in the most effective manner. In addition, coal mining enterprises should establish intelligent devices skills training courses and actively develop coal mine safety knowledge learning activities to improve coal miners’ comprehensive capabilities. They should develop a work system for regulating safety risks, and conduct timely accident hazard investigation and monitoring and maintenance of intelligent devices.

(3) Among the management factors, emergency management capability has the highest centrality. It is a dependent factor, making it necessary to improve the capacity of emergency preparedness, emergency disposal, and emergency recovery of unsafe accidents and reduce its influence by other factors. The leadership attention and safety investment levels are high driving and intermediate factors, and their degree of influence is high. The effect on other factors is significant, making it a core factor. It is necessary to focus on the control of these influencing factors, and this requires leadership’s attention to improve and promote intelligent equipment renewal, organization training, and other safety-related investment. Safety system normality is an intermediate factor and has a high degree of influence, making it necessary to attach great importance to improvement of embedded security systems. Coal mining enterprises should establish a sound, standardized system of coal mine safety and focus on occupational hazard prevention, accident reporting and accountability systems. Additionally, they should optimize the coal mining process in order to establish an efficient production model.

(4) The centrality of an organizational safety culture among the environmental factors is salient as it is a high-dependency factor. Thus, it is necessary to improve the organizational safety climate and promote organizational change and innovation. The degree of influence and causality of government supervision are the highest among all the influencing factors: this is a key driving factor that exerts the most significant influence on other factors. It proves that government supervision plays a crucial role in safety management under intelligent mining conditions, and it lies at the core of the influencing factor system. Government supervision should be increased to prevent and control safety accidents in the most effective way possible; moreover, a sound system of government supervision and safety responsibility should be established, with appropriate incentives and penalties to ensure safe production.

Different experts have different understandings and risk preferences for unsafe behaviors: they demonstrate different levels of theoretical knowledge and richness of practical experience, so expert weights can be introduced in the future to compensate for this deficiency. Meanwhile, the introduction of interval type-II fuzzy sets or the use of neural models instead of fuzzy logic to improve decision-making models deserves further exploration. In addition, the article is based on the example of an intelligent mine in China, where there are differences in safety policies and environments with regions such as Europe, which should further ensure coal mine safety and miners’ occupational health according to local regions’ regulations. Additionally, it needs to continuously improve the quality and application value of the research in combination the practical research analysis and risk assessment.

## Figures and Tables

**Table 1:** The fuzzy linguistic scale.

Linguistic Terms	Triangular Fuzzy Numbers
Very high influence (VH)	(0.75,1.0,1.0)
High influence (H)	(0.5,0.75,1.0)
Low influence (L)	(0.25,0.5,0.75)
Very low influence (VL)	(0,0.25,0.5)
No influence (No)	(0,0,0.25)

**Table 2:** Total relationship matrix.

	***x*_1_**	***x*_2_**	***x*_3_**	***x*_4_**	***x*_5_**	***x*_6_**	***x*_7_**	***x*_8_**	***x*_9_**	***x*_10_**
*x*_1_	0.0984	0.1600	0.1074	0.1573	0.1670	0.0810	0.0730	0.1378	0.1425	0.0973
*x*_2_	0.1679	0.1333	0.1224	0.1784	0.1952	0.0915	0.0850	0.1692	0.1915	0.1443
*x*_3_	0.1232	0.1091	0.0569	0.1271	0.1359	0.0633	0.0593	0.1046	0.1310	0.0827
*x*_4_	0.1195	0.1202	0.0686	0.0817	0.1160	0.0541	0.0532	0.1092	0.1049	0.0661
*x*_5_	0.1164	0.1441	0.0786	0.1467	0.1010	0.0678	0.0694	0.1435	0.1329	0.0780
*x*_6_	0.1358	0.1739	0.1026	0.1818	0.1829	0.0864	0.1098	0.1891	0.1870	0.1442
*x*_7_	0.1340	0.1722	0.0910	0.1765	0.1805	0.1247	0.0786	0.1842	0.1818	0.1488
*x*_8_	0.1689	0.1905	0.1194	0.1980	0.2121	0.1363	0.1281	0.1528	0.2025	0.1391
*x*_9_	0.1738	0.1889	0.1339	0.2035	0.2115	0.1405	0.1262	0.2103	0.1561	0.1687
*x*_10_	0.1604	0.1639	0.1138	0.1812	0.1765	0.1184	0.1111	0.1886	0.1953	0.1054
*x*_11_	0.2045	0.2299	0.1588	0.2366	0.2459	0.1813	0.1688	0.2464	0.2384	0.1790
*x*_12_	0.2035	0.2176	0.1483	0.2215	0.2395	0.1562	0.1413	0.2310	0.2289	0.1870
*x*_13_	0.2006	0.2334	0.1670	0.2437	0.2499	0.1724	0.1545	0.2505	0.2456	0.1958
*x*_14_	0.1786	0.2135	0.1404	0.2113	0.2260	0.1499	0.1448	0.2306	0.2252	0.1737
*x*_15_	0.1919	0.2067	0.1348	0.2003	0.2086	0.1275	0.1227	0.1975	0.1952	0.1499
*x*_16_	0.1567	0.1699	0.1558	0.1970	0.2016	0.1249	0.1239	0.1938	0.1832	0.1426
*x*_17_	0.1430	0.1580	0.1405	0.1813	0.1855	0.1196	0.1068	0.1697	0.1741	0.1399
*x*_18_	0.1958	0.2164	0.1583	0.2225	0.2224	0.1273	0.1226	0.2031	0.2115	0.1544
*x*_19_	0.2208	0.2399	0.1500	0.2346	0.2378	0.1817	0.1623	0.2382	0.2363	0.1857
*x*_20_	0.2033	0.2082	0.1485	0.2104	0.2188	0.1302	0.1039	0.1820	0.1990	0.1370
	***x*_11_**	***x*_12_**	***x*_13_**	***x*_14_**	***x*_15_**	***x*_16_**	***x*_17_**	***x*_18_**	***x*_19_**	***x*_20_**
*x*_1_	0.0936	0.1074	0.1042	0.1343	0.1649	0.0936	0.0970	0.1730	0.0694	0.1547
*x*_2_	0.1303	0.1601	0.1563	0.1705	0.1885	0.1334	0.1193	0.2008	0.0866	0.1807
*x*_3_	0.0723	0.0827	0.0768	0.1025	0.1244	0.0691	0.0721	0.1207	0.0568	0.1230
*x*_4_	0.0696	0.0925	0.0674	0.1016	0.1256	0.0612	0.0667	0.1225	0.0513	0.1273
*x*_5_	0.0980	0.0972	0.0844	0.1223	0.1332	0.0748	0.0876	0.1476	0.0632	0.1360
*x*_6_	0.1158	0.1565	0.1653	0.1552	0.1793	0.1119	0.1230	0.1891	0.0951	0.1660
*x*_7_	0.1144	0.1489	0.1610	0.1502	0.1866	0.1066	0.1184	0.1811	0.0978	0.1668
*x*_8_	0.1499	0.1789	0.1698	0.1922	0.2154	0.1397	0.1389	0.2131	0.1311	0.1962
*x*_9_	0.1536	0.1774	0.1772	0.1882	0.2148	0.1403	0.1464	0.2030	0.1177	0.1986
*x*_10_	0.1307	0.1672	0.1616	0.1738	0.1984	0.1239	0.1385	0.1803	0.1124	0.1773
*x*_11_	0.1374	0.2181	0.2182	0.2336	0.2559	0.1789	0.1895	0.2582	0.1445	0.2263
*x*_12_	0.1772	0.1546	0.2067	0.2254	0.2463	0.1816	0.1891	0.2394	0.1308	0.2240
*x*_13_	0.1755	0.2157	0.1574	0.2347	0.2567	0.1798	0.1903	0.2440	0.1344	0.2159
*x*_14_	0.1698	0.1954	0.1929	0.1580	0.2297	0.1713	0.1751	0.2289	0.1221	0.1937
*x*_15_	0.1591	0.1702	0.1796	0.1879	0.1656	0.1554	0.1552	0.2180	0.1191	0.1775
*x*_16_	0.1368	0.1526	0.1617	0.1783	0.2072	0.1013	0.1537	0.1891	0.1109	0.1831
*x*_17_	0.1279	0.1485	0.1546	0.1606	0.1910	0.1109	0.0984	0.1703	0.1009	0.1678
*x*_18_	0.1571	0.1963	0.1805	0.1938	0.2335	0.1535	0.1624	0.1697	0.0940	0.2080
*x*_19_	0.2023	0.2465	0.2228	0.2382	0.2669	0.1921	0.2029	0.2600	0.1071	0.2306
*x*_20_	0.1566	0.1796	0.1667	0.1886	0.2126	0.1499	0.1589	0.2134	0.1313	0.1507

**Table 3:** Centrality and degree of causality of factors.

Factor	*D*	*R*	*D* + *R*	*D* − *R*
*x*_1_	2.4138	3.2971	5.7110	−0.8833
*x*_2_	3.0054	3.6497	6.6551	−0.6444
*x*_3_	1.8936	2.4970	4.3906	−0.6034
*x*_4_	1.7791	3.7915	5.5706	−2.0124
*x*_5_	2.1225	3.9145	6.0370	−1.7920
*x*_6_	2.9506	2.4351	5.3857	0.5155
*x*_7_	2.9039	2.2450	5.1489	0.6590
*x*_8_	3.3727	3.7321	7.1048	−0.3594
*x*_9_	3.4310	3.7629	7.1938	−0.3319
*x*_10_	3.0785	2.8196	5.8982	0.2589
*x*_11_	4.1503	2.7280	6.8782	1.4223
*x*_12_	3.9501	3.2461	7.1962	0.7040
*x*_13_	4.1177	3.1651	7.2828	0.9526
*x*_14_	3.7312	3.4898	7.2211	0.2414
*x*_15_	3.4225	3.9967	7.4192	−0.5742
*x*_16_	3.2242	2.6292	5.8533	0.5950
*x*_17_	2.9492	2.7835	5.7327	0.1657
*x*_18_	3.5832	3.9223	7.5056	−0.3391
*x*_19_	4.2569	2.0766	6.3335	2.1803
*x*_20_	3.4495	3.6042	7.0537	−0.1547

**Table 4:** Threshold results by MMDE.

Item	Data
**Step 1:** The ordered $\mathrm{triplets}\;\mathrm{set}\;T^{*}$	$T^{*}$ = {(0.2669,19,15), (0.2600,19,18), (0.2582,11,18), (0.2567,13,15), (0.2559,11,15), …, (0.0513,4,19)}
$\mathrm{Step}\;2:\;T^{D_{i}}$$\mathrm{sets}\;\mathrm{and}\;T^{R_{e}}$ sets	$T^{D_{i}}$ = {19,19,11,13,11,…,4}
	$T^{R_{e}}$ = {15,18,18,15,15,…,19}
$\mathrm{Step}\;3.1:\;T_{t}^{D_{i}}$ sets	$T_{1}{}^{D_{i}}$$=\{19\};T_{2}{}^{D_{i}}$$=\{19,19\};\;T_{3}{}^{D_{i}}$$=\{19,19,11\};\;T_{4}{}^{D_{i}}$ = {19,19,11,13}; $T_{5}{}^{D_{i}}$$=\{19,19,11,13,11\};\;\ldots ;\;T_{400}^{R_{e}}$ = {19,19,11,13,11,…,4};
$\mathrm{Step}\;3.2:\;MDE_{t}^{D_{i}}$	{0,0.0283,0.0196,0.0146,0,…,0}
$\mathrm{Step}\;3.3:\;T_{t}^{R_{e}}$ sets	$T_{1}^{R_{e}}$$=\{15\};T_{2}^{R_{e}}$$=\{15,18\};T_{3}^{R_{e}}$$=\{15,18,18\};T_{4}^{R_{e}}$ = { 15,18,18,15}; $T_{5}^{R_{e}}$ $=\{15,18,18,15,15\};\ldots ;T_{400}^{R_{e}}$ = {15,18,18,15,15,…,19};
$\mathrm{Step}\;3.4:\;MDE_{t}^{R_{e}}$	{0,0,0.0283,0,0.0101,…,0}
$\mathrm{Step}\;4.1:\;\mathrm{Maximum}\;MDE_{t}^{D_{i}}$	0.0454
$\mathrm{Step}\;4.2:\;T_{\max}^{D_{i}}$	{11,12,13,19}
$\mathrm{Step}\;4.3:\;\mathrm{Maximum}\;MDE_{t}^{R_{e}}$	0.0291
$\mathrm{Step}\;4.4:\;T_{\max}^{R_{e}}$	{8,15,18}
**Step 5.1:** Dispatch-node $\mathrm{set}\;\mathrm{of}\;\mathrm{the}\;\mathrm{maximum}\;MDE_{t}^{D_{i}}$	{(0.2582,11,18),(0.2463,12,15),(0.2567,13,15),(0.2669,19,15)}
**Step 5.2:** Receive-node $\mathrm{set}\;\mathrm{of}\;\mathrm{the}\;\mathrm{maximum}\;MDE_{t}^{R_{e}}$	{(0.2505,13,8),(0.2669,19,15),(0.2600,19,18)}
$\mathrm{Step}\;5.3:\;T^{Th}$	{(0.2463,12,15),(0.2505,13,8),(0.2567,13,15), (0.2582,11,18),(0.2600,19,18),(0.2669,19,15),(0.2669,19,15)}
**Step 5.4:** Threshold value	0.2463

## Data Availability

Some empirical data of this study are presented in the Appendix A, Appendix B, Appendix C, Appendix D, Appendix E and Appendix F, and the rest can be obtained from the corresponding author upon reasonable request.

## Appendix A

**Table A1:** Data from the professor of mine safety management.

**Factor**	***x*_1_**	***x*_2_**	***x*_3_**	***x*_4_**	***x*_5_**	***x*_6_**	***x*_7_**	***x*_8_**	***x*_9_**	***x*_10_**
*x*_1_	NO	VH	VL	H	H	NO	NO	H	L	VL
*x*_2_	H	NO	NO	VL	VH	NO	NO	L	H	VL
*x*_3_	L	VL	NO	VL	L	NO	NO	NO	L	L
*x*_4_	H	H	NO	NO	L	NO	NO	VL	VL	NO
*x*_5_	H	VH	L	H	NO	NO	NO	L	L	NO
*x*_6_	VL	VH	VL	L	L	NO	VH	H	H	L
*x*_7_	L	H	NO	L	L	VH	NO	VH	H	L
*x*_8_	H	H	VL	L	H	H	VH	NO	L	L
*x*_9_	H	H	L	L	H	VH	VH	VH	NO	L
*x*_10_	L	L	L	L	L	H	H	H	VH	NO
*x*_11_	VH	VH	L	H	H	VH	VH	VH	H	H
*x*_12_	H	H	VL	L	H	H	H	H	H	H
*x*_13_	H	H	H	H	H	VH	VH	VH	VH	VH
*x*_14_	H	H	VL	L	H	VH	VH	VH	VH	VH
*x*_15_	H	H	VL	L	H	H	H	H	H	L
*x*_16_	L	VL	VH	H	H	H	H	H	L	L
*x*_17_	L	VL	VH	H	H	H	H	H	L	L
*x*_18_	VH	VH	H	VH	H	L	L	L	L	L
*x*_19_	H	H	L	H	H	H	H	H	H	H
*x*_20_	VH	H	H	H	H	VL	VL	VL	L	L
**Factor**	***x*_11_**	***x*_12_**	***x*_13_**	***x*_14_**	***x*_15_**	***x*_16_**	***x*_17_**	***x*_18_**	***x*_19_**	***x*_20_**
*x*_1_	L	L	VL	VL	H	L	L	VH	NO	H
*x*_2_	L	L	VL	L	H	VL	VL	VH	VL	H
*x*_3_	NO	NO	NO	NO	VL	NO	NO	VL	NO	L
*x*_4_	NO	NO	NO	NO	L	NO	NO	L	NO	L
*x*_5_	VL	L	NO	NO	VL	NO	NO	H	NO	H
*x*_6_	L	H	H	H	H	VL	NO	H	L	L
*x*_7_	L	L	H	H	H	NO	NO	L	L	VL
*x*_8_	L	H	H	H	H	VL	VL	H	H	H
*x*_9_	L	L	L	L	L	L	VL	L	VL	L
*x*_10_	VL	L	L	L	L	VL	VL	L	VL	L
*x*_11_	NO	VH	VH	VH	VH	H	H	VH	VL	H
*x*_12_	H	NO	VH	VH	VH	H	H	VH	VL	H
*x*_13_	L	H	NO	VH	VH	H	H	H	VL	H
*x*_14_	L	L	H	NO	H	L	L	H	VL	VL
*x*_15_	L	L	H	L	NO	L	L	H	VL	VL
*x*_16_	VL	VL	L	L	H	NO	L	L	NO	H
*x*_17_	VL	VL	L	L	H	NO	NO	L	NO	H
*x*_18_	L	VH	H	L	VH	VL	VL	NO	NO	VH
*x*_19_	VH	VH	VH	VH	VH	H	H	H	NO	H
*x*_20_	H	H	L	L	H	L	L	H	H	NO

## Appendix B

**Table A2:** Data from a miner in Shaanxi Coal Yubei Coal Industry Xiaobaodang Mining Co.

**Factor**	***x*_1_**	***x*_2_**	***x*_3_**	***x*_4_**	***x*_5_**	***x*_6_**	***x*_7_**	***x*_8_**	***x*_9_**	***x*_10_**
*x*_1_	NO	VL	L	VL	L	VH	H	VL	NO	H
*x*_2_	L	NO	L	VH	L	VL	VL	L	H	H
*x*_3_	VH	H	NO	L	H	H	H	L	L	L
*x*_4_	VL	VL	L	NO	VL	VL	L	VL	VL	L
*x*_5_	H	L	VL	H	NO	L	VH	H	VL	L
*x*_6_	H	H	H	L	L	NO	H	H	L	VL
*x*_7_	L	VH	VL	VL	L	VH	NO	VL	L	H
*x*_8_	VH	L	H	H	H	VH	H	NO	VH	H
*x*_9_	VH	VL	H	L	L	VH	H	VL	NO	VH
*x*_10_	H	H	L	L	H	L	L	VL	VL	NO
*x*_11_	VL	L	H	VL	H	L	VL	L	H	NO
*x*_12_	NO	VL	L	VL	L	VH	H	VL	NO	H
*x*_13_	L	VL	L	L	L	H	H	L	VL	L
*x*_14_	VL	H	H	VH	H	VH	L	VH	H	VL
*x*_15_	H	VH	H	L	VL	VL	L	H	H	NO
*x*_16_	VH	VH	VH	L	VL	VL	NO	H	NO	NO
*x*_17_	H	H	L	L	H	VL	H	H	L	VL
*x*_18_	L	H	NO	VL	L	H	L	L	VL	VL
*x*_19_	NO	L	VL	L	H	L	VL	NO	H	NO
*x*_20_	VH	H	L	VL	NO	H	NO	NO	L	NO
**Factor**	***x*_11_**	***x*_12_**	***x*_13_**	***x*_14_**	***x*_15_**	***x*_16_**	***x*_17_**	***x*_18_**	***x*_19_**	***x*_20_**
*x*_1_	NO	NO	L	NO	L	VL	VL	L	H	H
*x*_2_	NO	H	VH	L	VL	L	H	L	VL	VH
*x*_3_	H	H	L	VL	VH	H	H	L	H	VL
*x*_4_	H	H	NO	L	VL	VL	VL	L	L	H
*x*_5_	VL	L	VL	L	H	L	VL	H	H	NO
*x*_6_	VL	L	H	H	NO	H	L	L	H	H
*x*_7_	VL	VL	L	L	H	VH	L	L	H	VH
*x*_8_	L	VL	VL	L	H	H	NO	H	L	H
*x*_9_	H	L	H	L	H	VL	VL	L	L	H
*x*_10_	VL	L	H	VH	H	VL	L	H	L	L
*x*_11_	NO	VL	L	L	VL	VL	L	H	H	NO
*x*_12_	L	NO	H	L	L	H	VH	L	VL	H
*x*_13_	NO	H	NO	H	H	L	H	L	VL	NO
*x*_14_	L	H	VL	NO	VL	L	H	L	VL	VL
*x*_15_	L	NO	L	L	NO	H	L	H	L	VL
*x*_16_	L	VL	VL	L	H	NO	H	H	H	VL
*x*_17_	L	H	H	NO	L	L	NO	L	L	NO
*x*_18_	NO	L	NO	L	H	H	L	NO	VL	NO
*x*_19_	H	H	H	H	H	H	H	VH	NO	L
*x*_20_	NO	L	NO	L	VL	VL	L	H	H	NO

## Appendix C

**Table A3:** Defuzzified direct influence matrix.

	***x*_1_**	***x*_2_**	***x*_3_**	***x*_4_**	***x*_5_**	***x*_6_**	***x*_7_**	***x*_8_**	***x*_9_**	***x*_10_**
*x*_1_	0.0000	0.7800	0.5000	0.6867	0.7800	0.1933	0.1467	0.4533	0.4933	0.2533
*x*_2_	0.6867	0.0000	0.4400	0.5933	0.7800	0.0533	0.0533	0.5000	0.8267	0.6400
*x*_3_	0.6867	0.3600	0.0000	0.5933	0.6867	0.1467	0.1467	0.3000	0.6867	0.3000
*x*_4_	0.6867	0.5933	0.2067	0.0000	0.4533	0.0533	0.1000	0.4533	0.3600	0.1000
*x*_5_	0.4400	0.7333	0.2067	0.7333	0.0000	0.1000	0.1933	0.7333	0.5467	0.1000
*x*_6_	0.2533	0.6400	0.2000	0.6867	0.6400	0.0000	0.4400	0.8267	0.7800	0.6400
*x*_7_	0.2533	0.6400	0.0533	0.6400	0.6400	0.5800	0.0000	0.7800	0.7333	0.7333
*x*_8_	0.4933	0.5933	0.2533	0.6400	0.7800	0.5400	0.5333	0.0000	0.7333	0.3467
*x*_9_	0.5400	0.5467	0.4533	0.6867	0.7333	0.5867	0.4867	0.8267	0.0000	0.7800
*x*_10_	0.5467	0.4000	0.3000	0.5933	0.4467	0.3933	0.3933	0.7333	0.8267	0.0000
*x*_11_	0.5867	0.6867	0.4933	0.6867	0.7333	0.8733	0.8267	0.8733	0.7333	0.5400
*x*_12_	0.6867	0.6400	0.4067	0.5933	0.7800	0.5867	0.4933	0.7800	0.7333	0.7800
*x*_13_	0.5467	0.7800	0.6400	0.8267	0.8267	0.7733	0.6333	0.9667	0.8733	0.8267
*x*_14_	0.4467	0.7333	0.4000	0.5933	0.7333	0.5867	0.6333	0.9200	0.8267	0.6800
*x*_15_	0.8267	0.8267	0.4467	0.6400	0.6867	0.4000	0.4400	0.6400	0.5867	0.4933
*x*_16_	0.4067	0.4067	0.8733	0.7333	0.7333	0.4533	0.5467	0.7333	0.5467	0.5000
*x*_17_	0.3600	0.4067	0.7733	0.6867	0.6867	0.4933	0.4000	0.5467	0.5933	0.5933
*x*_18_	0.7733	0.8733	0.7333	0.8733	0.7800	0.3533	0.4000	0.6333	0.7333	0.4933
*x*_19_	0.7800	0.7800	0.3000	0.5933	0.5400	0.8267	0.6800	0.6800	0.6400	0.5867
*x*_20_	0.9667	0.8267	0.6400	0.7800	0.8267	0.4467	0.1600	0.4000	0.6400	0.3000
	***x*_11_**	***x*_12_**	***x*_13_**	***x*_14_**	***x*_15_**	***x*_16_**	***x*_17_**	***x*_18_**	***x*_19_**	***x*_20_**
*x*_1_	0.2000	0.2000	0.2067	0.4933	0.7333	0.2533	0.2533	0.8733	0.1467	0.7333
*x*_2_	0.4467	0.6400	0.6400	0.6400	0.6400	0.5467	0.2533	0.8733	0.1600	0.7333
*x*_3_	0.1467	0.1467	0.1000	0.3467	0.5000	0.1467	0.1467	0.4533	0.1467	0.5933
*x*_4_	0.1467	0.3467	0.0000	0.3933	0.5933	0.0533	0.1067	0.5467	0.1000	0.7333
*x*_5_	0.4067	0.2000	0.0533	0.4867	0.4533	0.1000	0.2533	0.6867	0.1467	0.6333
*x*_6_	0.2533	0.5933	0.7800	0.4400	0.5400	0.2533	0.3467	0.7333	0.3000	0.5467
*x*_7_	0.2533	0.5000	0.7333	0.3933	0.6867	0.1933	0.3000	0.6400	0.3533	0.5933
*x*_8_	0.5467	0.6867	0.5933	0.7333	0.7800	0.4533	0.3533	0.7800	0.6867	0.7333
*x*_9_	0.5933	0.6400	0.6867	0.6400	0.7333	0.4533	0.4533	0.5933	0.4533	0.7333
*x*_10_	0.4067	0.6867	0.6400	0.6400	0.7333	0.3600	0.5000	0.5000	0.5000	0.6400
*x*_11_	0.0000	0.8267	0.8733	0.8733	0.8267	0.6867	0.7333	0.9200	0.5933	0.6800
*x*_12_	0.6867	0.0000	0.8267	0.8733	0.8267	0.8267	0.8267	0.7800	0.4533	0.7800
*x*_13_	0.5867	0.8267	0.0000	0.9200	0.8733	0.7333	0.7800	0.7333	0.4533	0.5400
*x*_14_	0.6867	0.7333	0.7333	0.0000	0.7333	0.7800	0.7333	0.7800	0.4067	0.4533
*x*_15_	0.6867	0.5400	0.7333	0.6400	0.0000	0.6867	0.5933	0.8267	0.5000	0.4067
*x*_16_	0.4533	0.4067	0.5933	0.6400	0.7800	0.0000	0.6867	0.5467	0.4400	0.6400
*x*_17_	0.4533	0.5000	0.6400	0.5400	0.7333	0.2533	0.0000	0.4533	0.3933	0.5867
*x*_18_	0.5867	0.8733	0.6800	0.6400	0.9200	0.5933	0.6400	0.0000	0.0533	0.7733
*x*_19_	0.9200	0.9200	0.8733	0.8733	0.9200	0.8267	0.8733	0.8733	0.0000	0.6867
*x*_20_	0.6333	0.6867	0.5400	0.6400	0.6867	0.5933	0.6400	0.7333	0.6867	0.0000

## Appendix D

**Table A4:** Final reachability matrix.

**Factor**	***x*_1_**	***x*_2_**	***x*_3_**	***x*_4_**	***x*_5_**	***x*_6_**	***x*_7_**	***x*_8_**	***x*_9_**	***x*_10_**
*x*_1_	1	0	0	0	0	0	0	0	0	0
*x*_2_	0	1	0	0	0	0	0	0	0	0
*x*_3_	0	0	1	0	0	0	0	0	0	0
*x*_4_	0	0	0	1	0	0	0	0	0	0
*x*_5_	0	0	0	0	1	0	0	0	0	0
*x*_6_	0	0	0	0	0	1	0	0	0	0
*x*_7_	0	0	0	0	0	0	1	0	0	0
*x*_8_	0	0	0	0	0	0	0	1	0	0
*x*_9_	0	0	0	0	0	0	0	0	1	0
*x*_10_	0	0	0	0	0	0	0	0	0	1
*x*_11_	0	0	0	0	0	0	0	1	0	0
*x*_12_	0	0	0	0	0	0	0	0	0	0
*x*_13_	0	0	0	0	1	0	0	1	0	0
*x*_14_	0	0	0	0	0	0	0	0	0	0
*x*_15_	0	0	0	0	0	0	0	0	0	0
*x*_16_	0	0	0	0	0	0	0	0	0	0
*x*_17_	0	0	0	0	0	0	0	0	0	0
*x*_18_	0	0	0	0	0	0	0	0	0	0
*x*_19_	0	0	0	0	0	0	0	0	0	0
*x*_20_	0	0	0	0	0	0	0	0	0	0
**Factor**	***x*_11_**	***x*_12_**	***x*_13_**	***x*_14_**	***x*_15_**	***x*_16_**	***x*_17_**	***x*_18_**	***x*_19_**	***x*_20_**
*x*_1_	0	0	0	0	0	0	0	0	0	0
*x*_2_	0	0	0	0	0	0	0	0	0	0
*x*_3_	0	0	0	0	0	0	0	0	0	0
*x*_4_	0	0	0	0	0	0	0	0	0	0
*x*_5_	0	0	0	0	0	0	0	0	0	0
*x*_6_	0	0	0	0	0	0	0	0	0	0
*x*_7_	0	0	0	0	0	0	0	0	0	0
*x*_8_	0	0	0	0	0	0	0	0	0	0
*x*_9_	0	0	0	0	0	0	0	0	0	0
*x*_10_	0	0	0	0	0	0	0	0	0	0
*x*_11_	1	0	0	0	1	0	0	1	0	0
*x*_12_	0	1	0	0	1	0	0	0	0	0
*x*_13_	0	0	1	0	1	0	0	0	0	0
*x*_14_	0	0	0	1	0	0	0	0	0	0
*x*_15_	0	0	0	0	1	0	0	0	0	0
*x*_16_	0	0	0	0	0	1	0	0	0	0
*x*_17_	0	0	0	0	0	0	1	0	0	0
*x*_18_	0	0	0	0	0	0	0	1	0	0
*x*_19_	0	1	0	0	1	0	0	1	1	0
*x*_20_	0	0	0	0	0	0	0	0	0	1

## Appendix E

**Table A5:** Partitioning of reachability matrix.

**Factor**	**Reachability Set**	**Antecedent Set**	**Intersection Set**	**Rank**
*x*_1_	1	1	1	I
*x*_2_	2	2	2	I
*x*_3_	3	3	3	I
*x*_4_	4	4	4	I
*x*_5_	5	5, 13	5	I
*x*_6_	6	6	6	I
*x*_7_	7	7	7	I
*x*_8_	8	8, 11, 13	8	I
*x*_9_	9	9	9	I
*x*_10_	10	10	10	I
*x*_11_	8, 11, 15, 18	11	11	
*x*_12_	12, 15	12, 19	12	
*x*_13_	5, 8, 13, 15	13	13	
*x*_14_	14	14	14	I
*x*_15_	15	11, 12, 13, 15, 19	15	I
*x*_16_	16	16	16	I
*x*_17_	17	17	17	I
*x*_18_	18	11, 18, 19	18	I
*x*_19_	12, 15, 18, 19	19	19	
*x*_20_	20	20	20	I
**Factor**	**Reachability Set**	**Antecedent Set**	**Intersection Set**	**Rank**
*x*_11_	11	11	11	II
*x*_12_	12	12, 19	12	II
*x*_13_	13	13	13	II
*x*_19_	12, 19	19	19	III

## Appendix F

**Table A6:** Driving power and dependence power of factors.

Factor	Driving Power	Dependence Power	Factor	Driving Power	Dependence Power
*x*_1_	1	1	*x*_11_	4	1
*x*_2_	1	1	*x*_12_	2	2
*x*_3_	1	1	*x*_13_	4	1
*x*_4_	1	1	*x*_14_	1	1
*x*_5_	1	2	*x*_15_	1	5
*x*_6_	1	1	*x*_16_	1	1
*x*_7_	1	1	*x*_17_	1	1
*x*_8_	1	3	*x*_18_	1	3
*x*_9_	1	1	*x*_19_	4	1
*x*_10_	1	1	*x*_20_	1	1
